# Mitochondrial aberrations during the progression of disuse atrophy differentially affect male and female mice

**DOI:** 10.1002/jcsm.12809

**Published:** 2021-09-29

**Authors:** Megan E. Rosa‐Caldwell, Seongkyun Lim, Wesley S. Haynie, Jacob L. Brown, David E. Lee, Kirsten R. Dunlap, Lisa T. Jansen, Tyrone A. Washington, Michael P. Wiggs, Nicholas P. Greene

**Affiliations:** ^1^ Cachexia Research Laboratory, Exercise Science Research Center, Department of Health, Human Performance and Recreation University of Arkansas Fayetteville AR USA; ^2^ Exercise Muscle Biology Laboratory, Exercise Science Research Center, Department of Health, Human Performance and Recreation University of Arkansas Fayetteville AR USA; ^3^ Department of Health, Human Performance and Recreation Baylor University Waco TX USA

**Keywords:** Sex differences, Muscle, Catabolism, Mitophagy, Autophagy

## Abstract

**Background:**

Disuse decreases muscle size and is predictive of mortality across multiple pathologies. Detriments to mitochondrial function are hypothesized to underlie disuse‐induced muscle atrophy. Little data exist on early mechanisms contributing to onset of these pathologies, nor is it known how they differ between sexes. The purpose of this study was to examine differential and conserved responses to mitochondrial quality control in male and female mice during the development and progression of disuse‐induced atrophy.

**Methods:**

One hundred C57BL/6J mice (50 male and 50 female) were hindlimb unloaded to induce disuse atrophy for 0 (con), 24, 48, 72, or 168 h. At designated time‐points, extensor digitorum longus, gastrocnemius, and soleus muscles were collected for analysis of mitochondrial quality control markers.

**Results:**

One hundred sixty‐eight hours of disuse resulted in ~25% lower oxidative muscle fibre CSA in both male (*P* = 0.003) and female (*P* = 0.02) mice without any differences due to disuse in glycolytic fibres. In male mice, 48 h of unloading was sufficient to result in ~67% greater mitochondrial oxidative stress as assessed by the reporter gene pMitoTimer compared with 0 h (*P* = 0.002), this mitochondrial stress preceded detectable muscle loss. However in female mice, mitochondrial oxidative stress did not occur until 168 h of disuse (~40% greater mitochondrial oxidative stress in 168 h compared with 0 h of disuse, *P* < 0.0001). Blunted oxidative stress in female mice appeared to coincide with greater inductions of autophagy and mitophagy in female mice (~3‐fold greater BNIP3 and ~6‐fold greater LC3II/I ratio *P* < 0.0001 and *P* = 0.038 respectively). Male mice overall had greater reactive oxygen species (ROS) production compared with female mice. Female mice had a greater induction of ROS within 24 h of disuse (~4‐fold greater compared with 0 h, *P* < 0.0001); whereas male mice did not have greater ROS production until 168 h of disuse (~2‐fold greater, *P* < 0.0001). Although all muscle types exhibited some alterations to mitochondrial quality control, such as increased markers of mitophagy and fission, the soleus muscle in both male and female mice exhibited consistent alterations to various markers of mitochondrial quality. Markers of mitochondrial translation were approximately 30–50% lower within 24 h of unloading in both male and female soleus muscle (*P* value ranges: <0.0001–0.03).

**Conclusions:**

Disuse negatively affects mitochondria differentially between sexes during development of muscle wasting. Acutely, female mice may forgo muscle mass to maintain mitochondrial quality compared with male mice. These differences may contribute to divergent clinical manifestations of atrophy.

## Introduction

Disuse‐induced muscle loss is a frequent occurrence with spaceflight, casting of limbs, and prolonged bed rest.^1^ Prolonged lack of muscle contractile activity has significant implications for patients.^2^ Of note, disuse‐induced muscle atrophy is strongly associated with mortality in intensive care unit (ICU) patients.^3^ However, mechanisms contributing to this muscle loss and subsequent mortality are not fully understood.

Until recently, the breadth of muscle pathology research has predominantly been conducted using male models. Yet, recent works have begun to establish the importance of biological sex in relation to muscle pathologies.^4^ Clinically, many muscle pathologies present differently between male and female patients.^5^, ^6^ For example, female patients tend to have improved clinical outcomes and longevity compared with male patients during cancer cachexia development and progression.^7^ Conversely, during disuse atrophy, female patients tend to experience ICU frailty at higher rates compared with male patients,^8^ which appears to correspond to increased ICU mortality.^9^ Yet, preclinical models establishing mechanistic differences between male and female patients during muscle pathologies are limited, which limits the ability to develop therapeutics to mitigate muscle pathologies. Our recent work was the first to demonstrate biological sex differences in moderators of protein turnover between male and female patients during the early phases of disuse atrophy,^10^ although there are likely other mechanisms contributing to these sex‐related differences.

Current literature implicates mitochondrial quality as an important regulator of muscle health.^11^ Mitochondrial damage and dysfunction are common features among multiple myopathies such as disuse, cancer cachexia, muscular dystrophy, and sarcopenia.^12^, ^13^, ^14^, ^15^ We demonstrated mitochondrial degeneration precedes muscle loss in male mice during development of cancer cachexia,^16^ suggesting mitochondrial mechanisms as early alterations during the development muscle atrophy. Indeed, recent works have observed mitochondrial aberrations such as lowered respiration within 3 days of hindlimb unloading in female mice.^17^ However, whether these mitochondrial mechanisms are conserved between male and female mice remains to be investigated.

Overall, the aggregate of the literature suggests increased muscle mitochondrial dysfunction may underlie numerous muscle pathologies. However, the role of mitochondrial degeneration during the development of disuse‐induced muscle atrophy is not currently well‐defined. Furthermore, how mitochondrial dysfunction and quality control regulation differ between biological sexes is unknown. Therefore, the purpose of this study was to examine differential and conserved responses to mitochondrial quality in male and female mice during the development and progression of disuse‐induced atrophy.

## Methods

### Animals and interventions

Animal interventions were approved by The University of Arkansas Institutional Animal Care and Use Committee. One hundred C57BL6/J mice (50 male and 50 female, Jackson Laboratories, 000664) were randomly assigned to different periods of disuse atrophy [0 h (cage control; CON), 24 h, 48 h, 72 h, or 168 h with ~10 animals/group]. At 8 weeks of age animals underwent electroporation of the flexor digitorum brevis muscle with pMitoTimer as previously described^16^ and is described more thoroughly in *Data* S1. After 2 weeks of recovery, disuse atrophy was induced using the hindlimb unloading model.^10^ After 0–168 h of unloading, animals were euthanized and tissues collected. Animals were euthanized in a non‐fasted state, with *ad libitum* access to food and water until euthanasia. Because of differential response to disuse across different muscle phenotypes,^18^ we investigated mitochondrial aberrations across multiple muscle phenotypes including extensor digitorum longus (EDL, glycolytic muscle), gastrocnemius (mixed muscle), and soleus (oxidative muscle). A thorough description of hindlimb unloading and animal protocols can be found in our previous study using these same animals^10^ as well as *Data* S1.

### Mitochondrial respiration

Mitochondrial respiration in the soleus and gastrocnemius was performed as described^16^, ^19^ and is described in *Data* S1. The soleus and gastrocnemius were chosen to compare the effects of disuse atrophy on highly oxidative muscles with those on mixed muscles. Respiration was normalized to dry tissue weights and is presented as nmol/min * mg.

### Reactive oxygen species assay

Mitochondrial ROS emission from a permeabilized fibre bundle from the soleus and gastrocnemius were determined using Amplex Red™ reagent (Life Technologies)^16^ and described in *Data* S1.

### Histological analysis

#### pMitoTimer

pMitoTimer of the flexor digitorum brevis was analyzed as we have described^16^ and is thoroughly described in *Data* S1. All slides were analyzed at time of harvest for the ratio of red/green fluorescence, whereby greater red/green ratios are indicative of increased mitochondrial stress.^20^ Slides were also measured for pure red puncta, a marker for completely degenerated mitochondria.^20^ All images were acquired at 100× magnification using a Nikon TiS epifluorescent microscope. Acquisition and image analysis parameters were kept consistent across all groups.

For additional histological analysis, portions of the tibialis anterior muscle from each animal were frozen in optimal cutting medium submerged in cooled isopentane. Sections of the tibialis anterior were cut 10 μm thick using a Leica CM1860 (Leica Biosystems, Buffalo Grove, IL, USA) cryostat microtome. Samples were stained for succinate dehydrogenase (SDH) and periodic acid–Schiff stain. Further details can be found in *Data* S1.

### mRNA analysis

We analyzed mRNA content in the EDL (predominantly fast twitch fibre), Gastrocnemius (mixed fibre type), and soleus (predominantly oxidative fibres) muscles. Tissue homogenization, RNA isolation, cDNA synthesis, and quantitative reverse transcription PCR analysis are described in *Data* S1. All samples were normalized to 18 s, which did not differ between groups.

### Western blot analysis

Western blot analysis of the gastrocnemius and EDL muscles were completed as we have described.^21^ Antibodies included COX‐IV (Cell Signaling 4844S), PGC‐1α (Novus Biologicals, NBP1‐04676SS), MFN1 (Santa Cruz sc‐50330), MFN2 (Santa Cruz sc‐50331), OPA1 (Santa Cruz sc‐367890), DRP1 (Cell Signaling 14647), FIS1 (Novus NB100‐56646). BNIP3 (Cell Signaling 3769), LC3 (Cell Signaling 4108), p62 (Cell Signaling 5114s). Western blot analysis was only performed in gastrocnemius and EDL tissues due to tissue availability.

### Statistical analysis

A one‐way ANOVA with Tukey *post‐hoc* test was used within each biological sex with factor levels including hours of disuse (0, 24, 48, 72, or 168 h). Additionally, to determine how outcome variables are altered during the progression of disuse we utilized trend analysis as we have previously published and described.^10^ This analysis was used to determine overall patterns of the data that may not reach pairwise statistical significance and indirectly compare differential progressions of disuse atrophy in male and female mice.

## Results

### Hindlimb unloading resulted in decreased muscle size in both sexes

**Figure 1:**
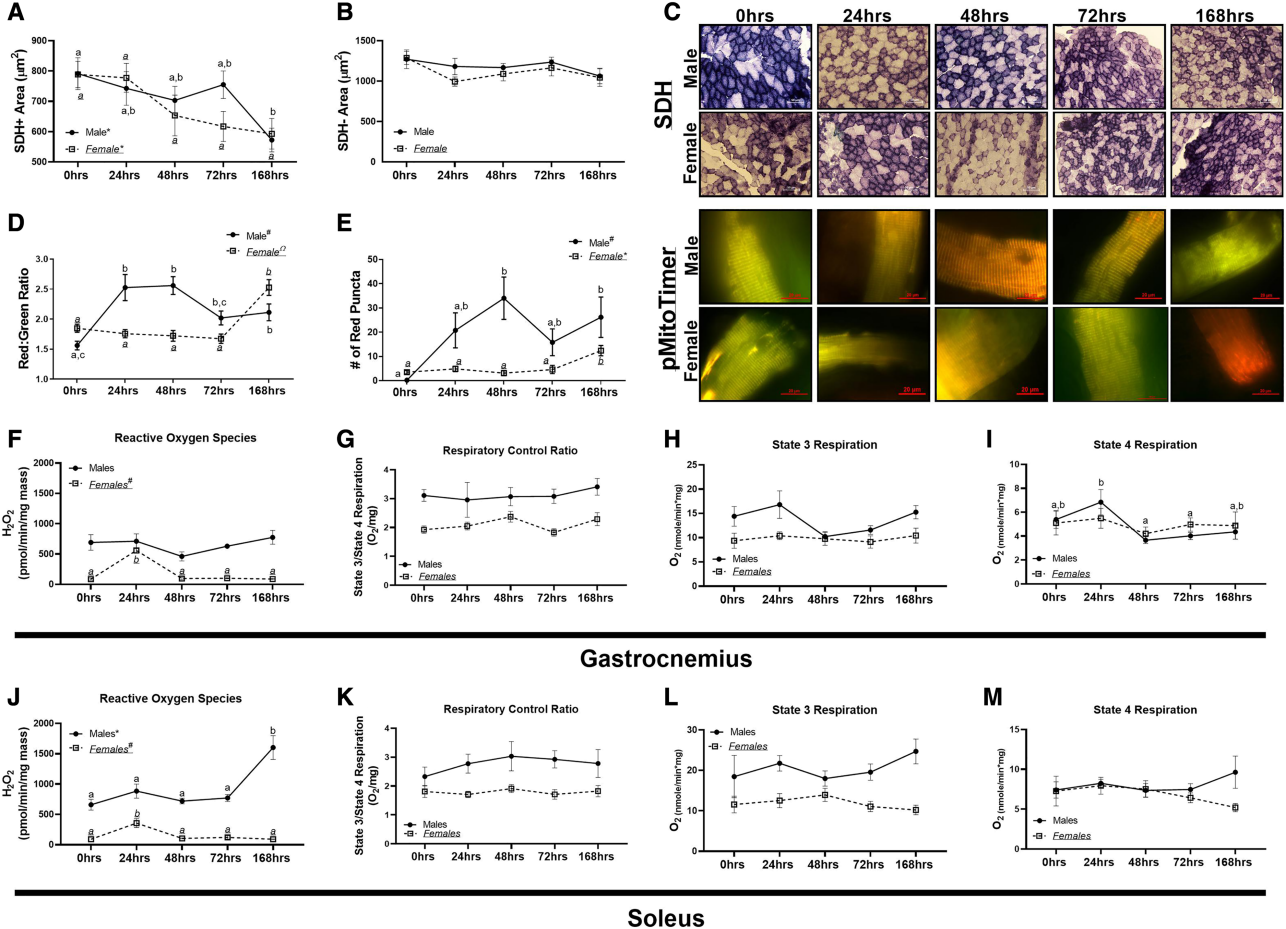
Measures of muscle metabolic alterations and mitochondrial function across disuse atrophy. (A) Cross‐sectional area of SDH + muscle fibres. (B) Cross‐sectional area of SDH− muscle fibres. (C) Representative images for SDH and pMitoTimer in male and female mice. (D) pMitotimer red:green ratio in male and female mice. (E) Red puncta content in male and female mice. (F) Reactive Oxygen Species (ROS) production in the gastrocnemius of male and female mice. (G) Respiratory exchange ratio (RCR) of State 3 and State 4 respiration in the gastrocnemius muscle in male and female mice. (H) State 3 respiration in the gastrocnemius muscle in male and female mice. (I) State 4 respiration in the gastrocnemius muscle in male and female mice. (J) ROS production in the soleus of male and female mice. (K) RCR of State 3 and State 4 respiration in the soleus muscle in male and female mice. (L) State 3 respiration in the soleus muscle in male and female mice. (M) State 4 respiration in the soleus muscle in male and female mice. Different letters indicate statistical differences within a sex at *P* < 0.05. * indicates linear trend, Ω indicates quadratic trend, and # indicates cubic trend within as sex.

We previously reported raw tissue weights in these animals demonstrating reductions in muscle size within 24–48 h of hindlimb unloading.^10^ Female mice had significantly lower body and muscle weights within 24 h of unloading while male mice did not present with muscle atrophy until 48 h of unloading.^10^ Male mice demonstrated a linear decrease in SDH + CSA (*P* = 0.003, *Figure* 1A and 1C). Similarly, female mice experienced a linear trend for lower CSA of SDH + muscle fibres (*P* = 0.02, *Figure* 1A and 1C). However, neither male nor female mice exhibited differences in CSA of SDH− muscle fibres (*P* = 0.51 and *P* = 0.08 respectively, *Figure* 1B and 1C, *P* > 0.05). Frequency histograms for SDH + and SDH− fibres as well as intramuscular glycogen content can be found in Supporting information, *Figure* S1. Mitochondrial content measured by *Cox4* mRNA and COXIV protein did not differ between time points in either male or female mice (*Figure* S2). Measures of oxidative metabolism, *Pparα*, *Nrf2*, and *Pgc1α* demonstrated some moderate aberrations with HU across muscle types (*Figure* S3).

In male mice, red:green ratio was approximately 65% greater after 24 and 48 h of disuse (quadratic relationship, *P* < 0.002, *Figure* 1D and 1C). Contrastingly, in female mice, a linear relationship was noted, with greater durations of unloading associated with greater red:green ratio (*P* < 0.0001, *Figure* 1D and 1C). Red puncta, a measure of degenerated mitochondria tagged for degeneration,^20^ demonstrated a quadratic trend in male mice, with 30‐fold greater red puncta at 24 and 48 h of disuse (*P* < 0.05), whereas female mice did not have alterations in red puncta until 168 h of disuse (linear trend *P* < 0.0001, *Figure* 1E and 1C). There was no difference in ROS generation in male gastrocnemius muscle (*P* = 0.269); although female mice demonstrated a quadratic trend (*P* < 0.0001, *Figure* 1F). Mitochondrial respiratory control ratio (RCR) was not different in male or female gastrocnemius (*P* = 0.951 and *P* = 0.113, respectively; *Figure* 1G). State 3 respiration was not different in either male or female gastrocnemius muscle (*P* = 0.063 and *P* = 0.926, respectively; *Figure* 1H). State 4 respiration in male gastrocnemius muscle was significantly different between 24, 48, and 72 h animals (*Figure* 1I), whereas there were no differences or trends noted in female gastrocnemius muscle (*P* = 0.877, *Figure* 1I).

In the soleus muscle, male mice demonstrated a linear trend for ROS generation (*P* < 0.0001, *Figure* 1J), whereas in female soleus muscle, a quadratic trend was noted (*P* < 0.0001, *Figure* 1J). Neither male nor female mice had any differences noted in RCR in the soleus muscle (*P* = 0.782 and *P* = 0.113, respectively, *Figure* 1K). Correspondingly, there were no differences in either male or female mice in State 3 (*P* = 0.597 and *P* = 0.561, respectively, *Figure* 1L) or State 4 respiration (*P* = 0.546 and *P* = 0.448, *Figure* 1M).

### Markers of mitochondrial mRNA translation demonstrated muscle specific alterations during the duration of hindlimb unloading

**Figure 2:**
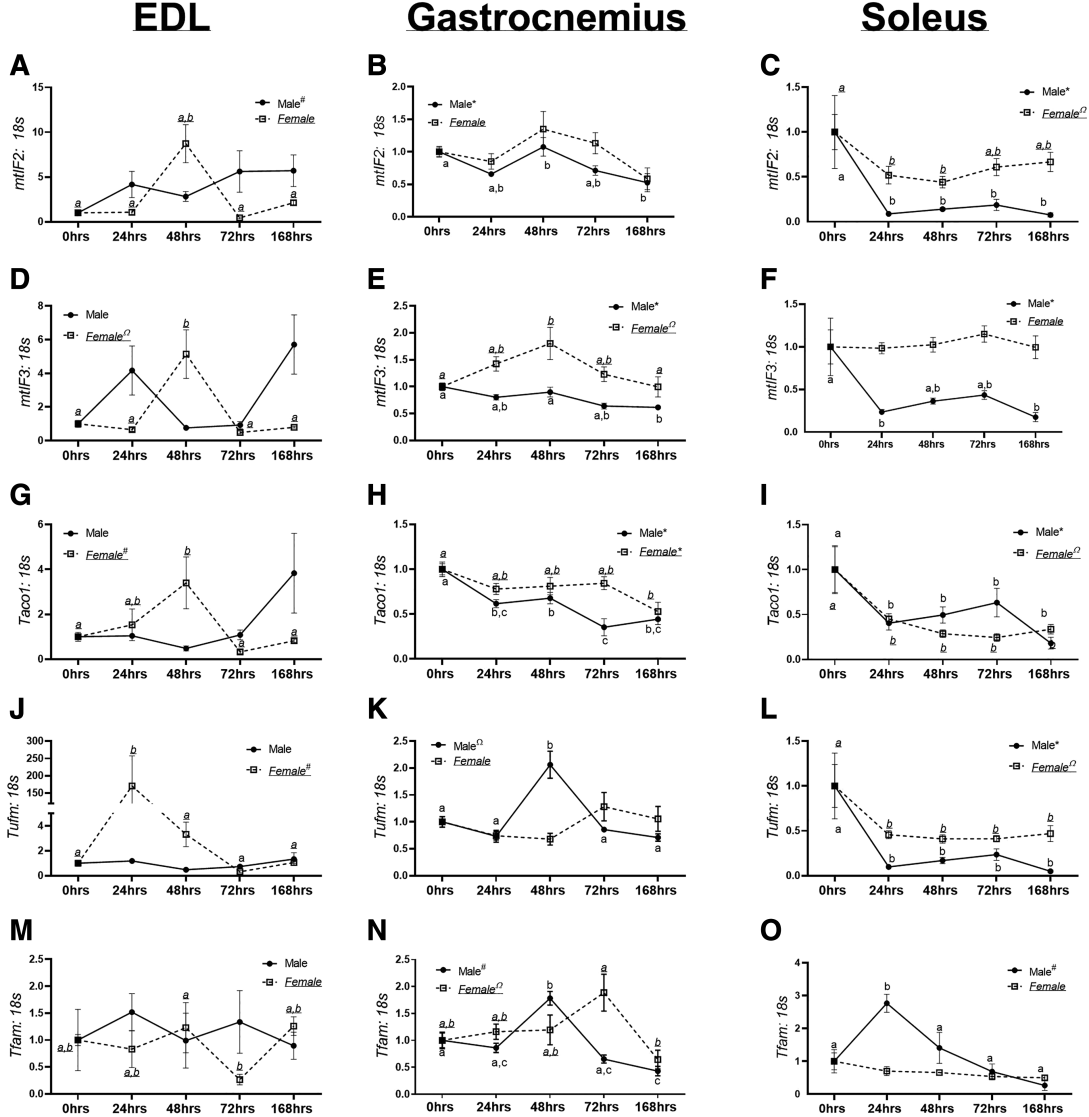
mRNA content of markers of mitochondrial translation. (A) *mtiF2* mRNA content in extensor digitorum longus (EDL) muscle in male and female mice. (B) *mtiF2* mRNA content in gastrocnemius muscle in male and female mice. (C) *mtiF2* mRNA content in the soleus muscle in male and female mice. (D) *mtiF3* mRNA content in EDL muscle in male and female mice. (E) *mtiF3* mRNA content in the gastrocnemius muscle in male and female mice. (F) *mtiF3* mRNA content in the soleus muscle in male and female mice. (G) *Taco1* mRNA content in the EDL muscle in male and female mice. (H) *Taco1* mRNA content in the gastrocnemius muscle in male and female mice. (I) *Taco1* mRNA content in the soleus muscle in male and female mice. (J) *Tufm* mRNA content in the EDL muscle in male and female mice. (K) *Tufm* mRNA content in the gastrocnemius muscle in male and female mice. (L) *Tufm* mRNA content in the soleus muscle in male and female mice. (M) *Tfam* mRNA content in the EDL muscle in male and female mice. (N) *Tfam* mRNA content in the gastrocnemius muscle in male and female mice. (O) *Tfam* mRNA content in the soleus muscle in male and female mice. Different letters indicate statistical differences within a sex at *P* < 0.05. * indicates linear trend, Ω indicates quadratic trend, and # indicates cubic trend within as sex.

We also measured markers of mitochondrial mRNA translation, the system for mRNA translation of mitochondrial DNA encoded proteins that are imperative for oxidative phosphorylation.^17^ In the EDL muscle, there were no differences noted in male *mtiF2* content (*P* = 0.166, *Figure* 2A). However, in female EDL muscles, a cubic trend was noted in *mtiF2* (*P* < 0.006, *Figure* 2A). In the gastrocnemius muscle, a linear trend was found in male mice (*P* < 0.006, *Figure* 2B), but not in female mice (*P* = 0.063, *Figure* 2B). In the soleus muscle, both male and female mice exhibited lower *mtiF2* content in all HU groups compared with 0 h control (*P* < 0.006 and *P* < 0.02, respectively, *Figure* 2C).

In the EDL muscle, no trends were noted for *mtifF3* in male mice (*P* = 0.16, *Figure* 2D). In female EDL muscles, a quadratic trend was noted in *mtifF3* content (*P* < 0.04, *Figure* 2D). *mtIF3* mRNA content in male gastrocnemius muscle demonstrated a linear trend (*P* < 0.0001, *Figure* 2E). In female gastrocnemius muscle, a quadratic trend was noted in *mtiF3* (*P* < 0.03, *Figure* 2E). In the soleus muscle, male mice had a linear decrease in *mtifF3* content (*P* < 0.02, *Figure* 2F), whereas there were no differences noted in female mice.

Within the EDL muscle, male mice did not have any differences or trends noted in *Taco1* content (*P* = 0.07, *Figure* 2G). In female mice, a cubic trend was noted (*P* < 0.02, *Figure* 2G). Within the gastrocnemius muscle, a linear trend was found in *Taco1* content in both male and female mice (*P* < 0.05 and *P* < 0.0008, respectively, *Figure* 2H). Within the soleus muscle, male and female mice had linear decreases in *Taco1* content (*P* < 0.005 and *P* < 0.005, respectively, *Figure* 2I).

In the EDL muscle, male mice did not have any differences or trends in *Tufm* content (*P* = 0.22, *Figure* 2J). However female mice had a cubic trend noted for *Tufm* content (*P* < 0.005, *Figure* 2J). Within the gastrocnemius muscle, male mice had a quadratic trend noted in *Tufm* content (*P* < 0.003, *Figure* 2K), whereas there were no differences noted in female mice (*P* = 0.10, *Figure* 2K). In the soleus muscle, male and female mice had lower *Tufm* content (*P* < 0.007 and *P* < 0.03, *Figure* 2L), with a linear trend noted in male mice and quadratic trend noted in female mice.

In male and female EDL muscle, no trends or differences were noted for *Tfam* (*P* = 0.86 and *P* = 0.31, *Figure* 2M). In the gastrocnemius muscle, male and female mice had quadratic trends in *Tfam* content (*P* < 0.008 and *P* < 0.006, *Figure* 2N). In the soleus muscle, male mice had a significant cubic trend in *Tfam* content (*P* < 0.0002, *Figure* 2O). In female mice, no trends or differences were noted in *Tfam* content (*P* = 0.14, *Figure* 2O).

### Mitochondrial dynamics were altered during the progression of hindlimb unloading

**Figure 3:**
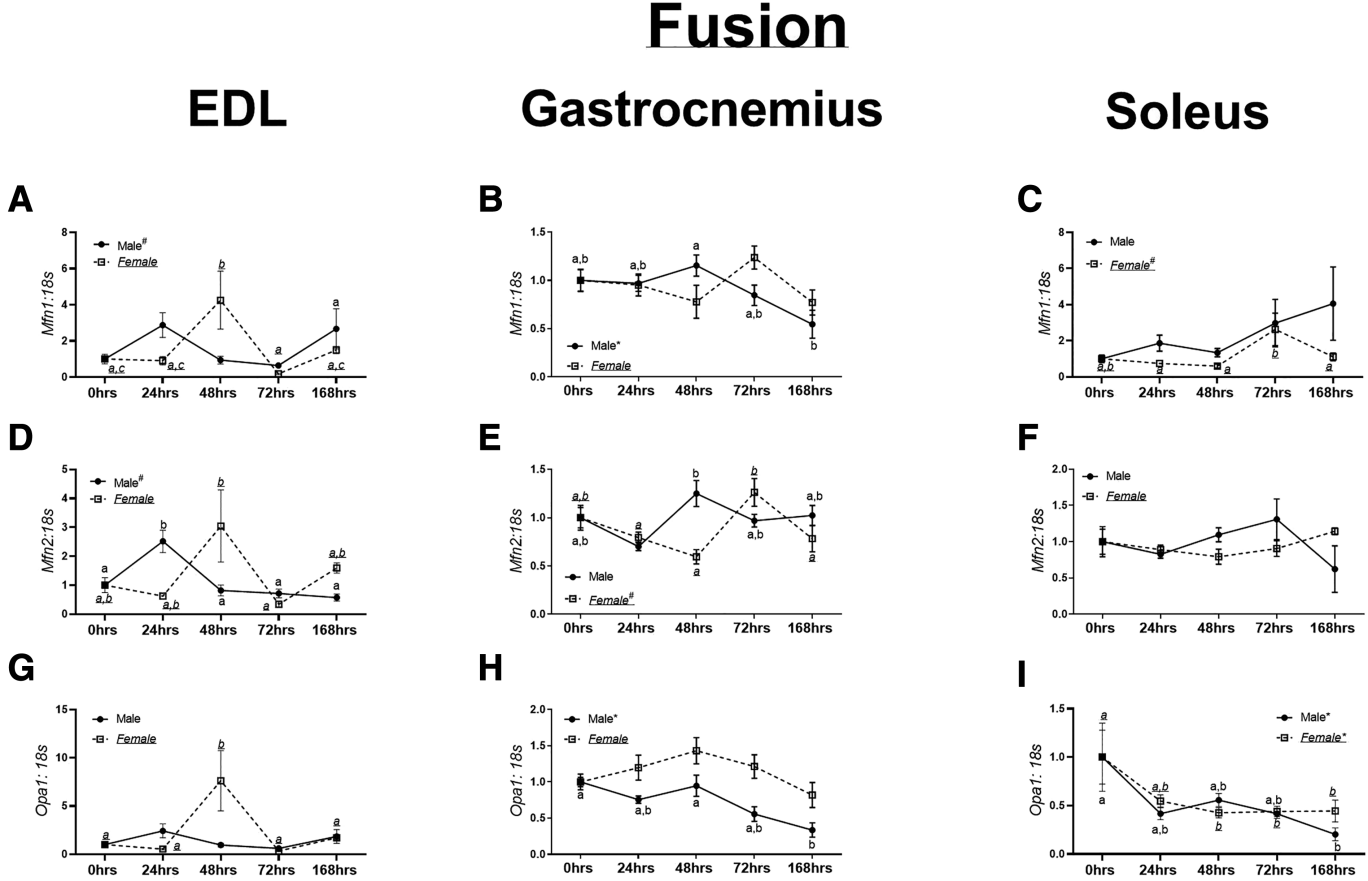
mRNA and protein content of markers of mitochondrial fusion. (A) *Mfn1* mRNA content in the extensor digitorum longus (EDL) muscle in male and female mice. (B) *Mfn1* mRNA content in the gastrocnemius muscle in male and female mice. (C) *Mfn1* mRNA content in the soleus muscle in male and female mice. (D) *Mfn2* mRNA content in the EDL muscle in male and female mice. (E) *Mfn2* mRNA content in the gastrocnemius muscle in male and female mice. (F) *Mfn2* mRNA content in the soleus muscle in male and female mice. (G) *Opa1* mRNA content in the EDL muscle in male and female mice. (H) *Opa1* mRNA content in the gastrocnemius muscle in male and female mice. (I) *Opa1* mRNA content in the soleus muscle in male and female mice. Different letters indicate statistical differences within a sex at *P* < 0.05. * indicates linear trend, Ω indicates quadratic trend, and # indicates cubic trend within as sex.

In the EDL muscle, there were no trends or differences noted in *Mfn1* mRNA content in male mice (*P* = 0.06, *Figure* 3A). In female EDL muscle, no trends were noted (*P* = 0.08–0.9, *Figure* 3A). Male gastrocnemius muscle had a linear trend towards lowered *Mfn1* (*P* < 0.002, *Figure* 3B). In female gastrocnemius muscle no trends or differences were noted in *Mfn1* content (*P* = 0.11, *Figure* 3B). In male soleus muscle, there were no trends in *Mfn1* content (*P* = 0.28, *Figure* 3C). However, in female soleus muscle, a cubic trend was found (*P* < 0.006, *Figure* 3C).

In male EDL muscle, there was a cubic trend noted in *Mfn2* content (*P* < 0.003, *Figure* 3D), although no trends noted in female EDL (*P* = 0.31, *Figure* 3D). Within male gastrocnemius muscle, a global *F* test was noted in *Mfn2* content (*P* = 0.02, *Figure* 5E); however, no trends were noted. In female gastrocnemius muscle, a cubic trend was noted in *Mfn2* (*P* < 0.0006, *Figure* 3E). No differences were noted in *Mfn2* content in the soleus muscle of either male or female mice (*P* = 0.22 and *P* = 0.28, *Figure* 3F).

In male EDL muscle, no differences or trends were found in *Opa1* content (*P* = 0.07, *Figure* 3G). In female mice, 48 h animals had approximately 5–7‐fold greater *Opa1* mRNA content compared with 0 h (*P* = 0.002, *Figure* 3G). Within male gastrocnemius, a linear trend was detected (*P* < 0.0001, *Figure* 3H). Contrastingly, in female gastrocnemius muscle, no trends were noted in *Opa1* mRNA content (*P* = 0.09, *Figure* 3H). In male and female soleus, a linear trend was noted in *Opa1* content (*P* = 0.005 and *P* = 0.03, *Figure* 3I). MFN1, MFN2, and OPA1 Western blot data are described in *Data* S2 and depicted in *Figure* S4.

**Figure 4:**
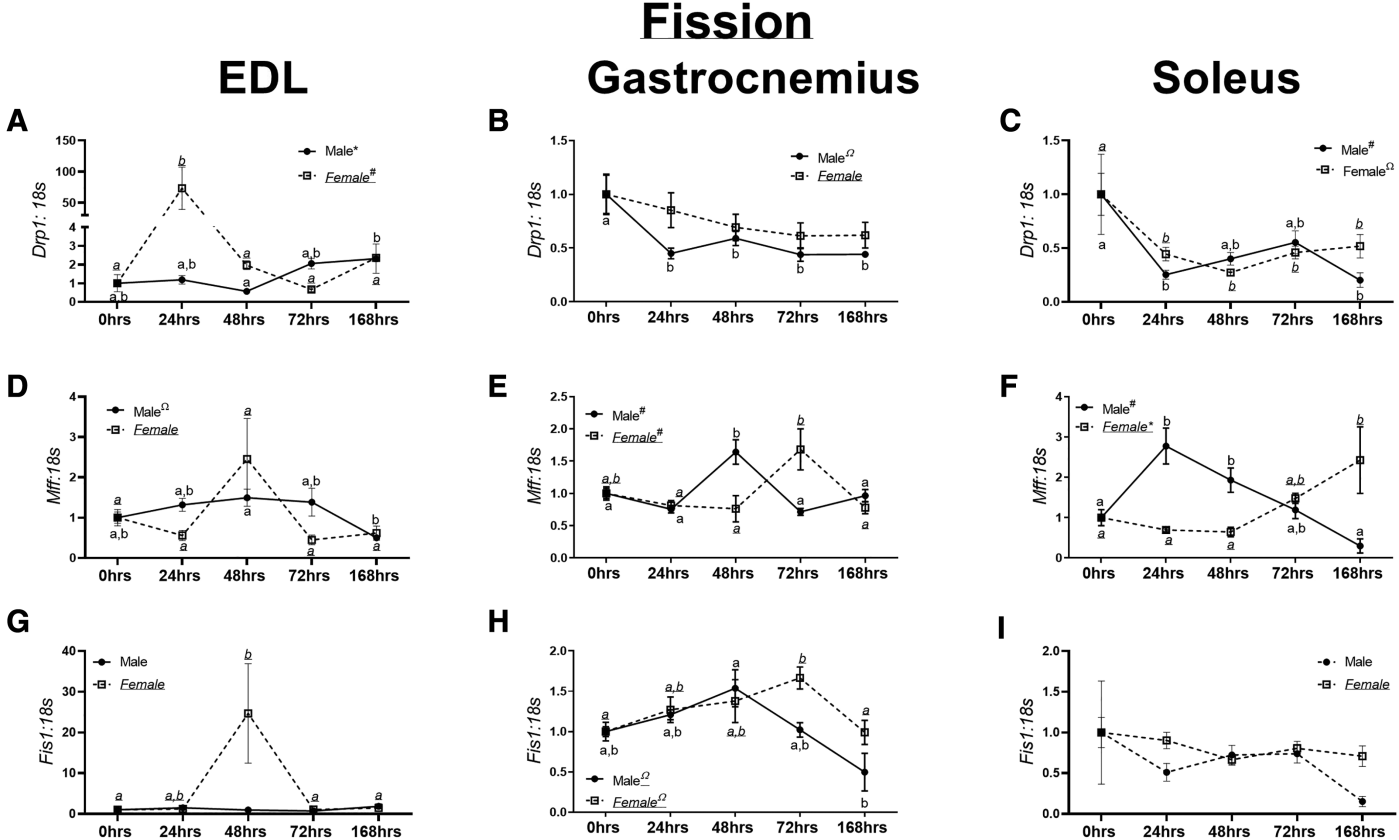
mRNA and protein content of markers of mitochondrial fission. (A) *Drp1* mRNA content in the extensor digitorum longus (EDL) muscle in male and female mice. (B) *Drp1* mRNA content in the gastrocnemius muscle in male and female mice. (C) *Drp1* mRNA content in the soleus muscle in male and female mice. (D) *Mff* mRNA content in the EDL muscle in male and female mice. (E) *Mff* mRNA content in the gastrocnemius muscle in male and female mice. (F) *Mff* mRNA content in the soleus muscle in male and female mice. (G) *Fis1* mRNA content in the EDL muscle in male and female mice. (H) *Fis1* mRNA content in the gastrocnemius muscle in male and female mice. (I) *Fis1* mRNA content in the soleus muscle in male and female mice. Different letters indicate statistical differences within a sex at *P* < 0.05. * indicates linear trend, Ω indicates quadratic trend, and # indicates cubic trend within as sex.

Male EDL muscle demonstrated a linear trend in *Drp1* content (*P* < 0.01, *Figure* 4A), whereas a cubic trend was found in female EDL muscle (*P* < 0.002, *Figure* 4A). With male gastrocnemius muscle, a linear decrease in *Drp1* content was noted (*P* < 0.005, *Figure* 4B), although no trends were found in female gastrocnemius muscle (*P* = 0.30, *Figure* 4B). In male soleus, a linear trend was noted in *Drp1* content (*P* < 0.02, *Figure* 4C). In female soleus, a quadratic trend was noted (*P* < 0.0002, *Figure* 4A).

Within male EDL muscle, a quadratic trend was observed in *Mff* content (*P* < 0.02, *Figure* 4D), with no differences found in female EDL muscle (*P* = 0.06, *Figure* 4D). In male and female gastrocnemius muscle, cubic trends were noted in *Mff* content (*P* < 0.05 and *P* < 0.003, *Figure* 4E). Within the soleus muscle of male mice, a cubic trend was noted in *Mff* content (*P* < 0.0001, *Figure* 4F). In female mice, a linear trend was noted in *Mff* content (*P* < 0.0005, *Figure* 4F).

In male EDL muscle, no trends were observed in *Fis1* content (*P* = 0.21, *Figure* 4G). However, in female EDL muscle, a quadratic trend was noted in *Fis1* content (*P* < 0.02, *Figure* 4G). Within the gastrocnemius muscle of male mice, a linear trend was detected in *Fis1* (*P* < 0.005, *Figure* 4H). Contrastingly, in female gastrocnemius muscle, a quadratic trend was noted in *Fis1* content (*P* < 0.002, *Figure* 4H). Within the soleus muscle, no differences or trends were noted in *Fis1* content in either male or female mice (*P* = 0.28 and *P* = 0.25 respectively, *Figure* 4I). DRP1 and FIS1 Western blot data are described in *Data* S2 and depicted in *Figure* S4.

### Mitophagy demonstrated dimorphic responses between male and female mice

**Figure 5:**
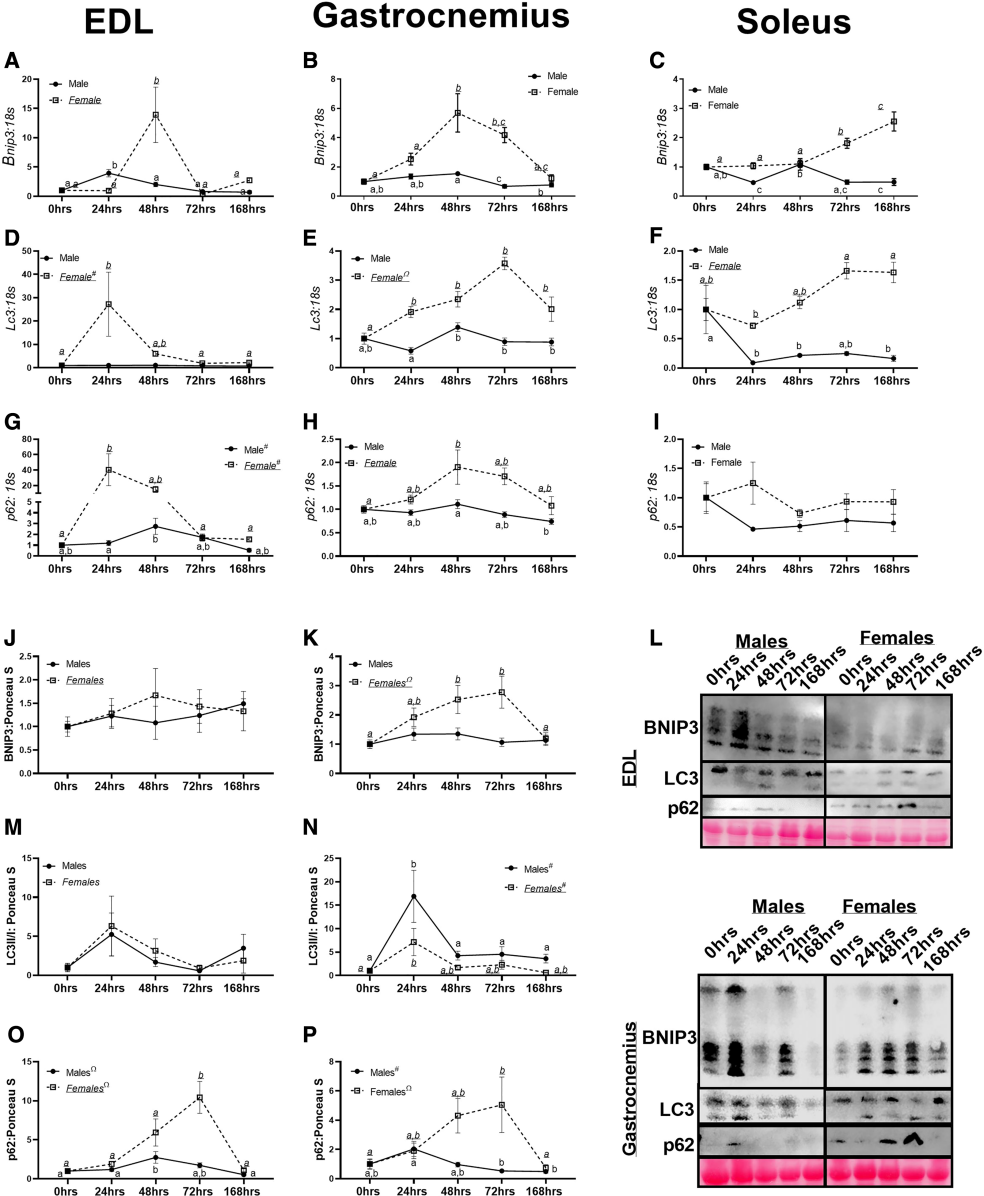
mRNA and protein content of markers of mitophagy and autophagy. (A) *Bnip3* mRNA content in the extensor digitorum longus (EDL) muscle in male and female mice. (B) *Bnip3* mRNA content in the gastrocnemius muscle in male and female mice. (C) *Bnip3* mRNA content in the soleus muscle in male and female mice. (D) *Lc3* mRNA content in the EDL muscle in male and female mice. (E) *Lc3* mRNA content in the gastrocnemius muscle in male and female mice. (F) *Lc3* mRNA content in the soleus muscle in male and female mice. (G) *p62* mRNA content in the EDL muscle in male and female mice. (H) *p62* mRNA content in the gastrocnemius muscle in male and female mice. (I) *p62* mRNA content in the soleus muscle in male and female mice. (J) BNIP3 protein content in the EDL muscle in male and female mice. (K) BNIP3 protein content in the gastrocnemius muscle in male and female mice. (L) Representative images of Western Blot images for EDL and gastrocnemius muscles. (M) LC3II/I protein content in the EDL muscle in male and female mice. (N) LC3II/I protein content in the gastrocnemius muscle in male and female mice. (O) p62 protein content in the EDL muscle in male and female mice. (P) p62 protein content in the gastrocnemius muscle in male and female mice. Different letters indicate statistical differences within a sex at *P* < 0.05. * indicates linear trend, Ω indicates quadratic trend, and # indicates cubic trend within as sex.

In male and female EDL muscle, cubic trends were noted in *Bnip3* content (*P* < 0.0001 and *P* < 0.008, *Figure* 5A). Within male gastrocnemius muscle, a cubic trend was noted in *Bnip3* content (*P* < 0.0003, *Figure* 5B). However, in female gastrocnemius muscle, *Bnip3* appeared to have more robust alterations with hindlimb unloading, with a quadratic trend noted (*P* < 0.0001, *Figure* 5B). Within male soleus muscle, *Bnip3* demonstrated a linear decrease (*P* < 0.01, *Figure* 5C). Contrastingly, female mice had a linear increase in *Bnip3*, with longer durations of unloading corresponding to greater *Bnip3* content (*Figure* 5C). There were no differences noted in EDL BNIP3 content in either male or female mice (*P* = 0.732 and *P* = 0.852, respectively, *Figure* 5J and 5L). In male gastrocnemius muscle, there were no trends of pairwise differences detected in BNIP3 content (*P* = 0.552, *Figure* 5K and 5L). However in female mice, a quadratic trend was detected (*P* < 0.001, *Figure* 5K and 5L), whereby 48 and 72 h animals had approximately 2.7‐fold greater BNIP3 compared to 0 and 168 h animals.

No differences or trends in *Lc3* content were observed in male EDL muscle (*P* = 0.33, *Figure* 5D). However in female EDL muscle, a cubic trend was observed in *Lc3* content (*P* < 0.003, *Figure* 5D). Within male gastrocnemius muscle, 48 h male mice had approximately 50–80% greater *Lc3* content compared with 24, 72, and 168 h animals (*Figure* 5E). Contrastingly, in female gastrocnemius muscle a quadratic trend was detected (*P* < 0.0001, *Figure* 5E). In the soleus muscle, male mice demonstrated a quadratic relationship with regards to *Lc3* content (*P* < 0.05, *Figure* 5F). There were no trends noted in LC3II/I ratio in either male or female EDL muscle (*P* = 0.160 and *P* = 0.510, respectively, *Figure* 5M and 5L). In male gastrocnemius muscle, a significant cubic trend (*P* = 0.011, *Figure* 5N and 5L) was found in LC3II/I ratio. A similar pattern was noted in LC3II; however, there was no statistical significance (*P* = 0.081, *Figure* S5). In female gastrocnemius muscle, a cubic trend was also noted in LC3II/I content (*P* = 0.038, *Figure* 5N and 5L) with 24 h animals having approximately 6‐fold greater LC3II/I content compared with 0 h animals.

In male EDL muscle, a linear trend was observed in *p62* content (*P* = 0.0065, *Figure* 5G). Contrastingly, in female mice, a cubic trend was noted in *p62* content (*P* < 0.002, *Figure* 5G). Within male gastrocnemius muscle, a linear decrease in *p62* content was detected (*P* < 0.003, *Figure* 5H). In female gastrocnemius muscle, a quadratic trend was noted (*P* < 0.002, *Figure* 5H). No statistical differences were noted in *p62* content in either male or female soleus muscles (*P* = 0.63 and *P* = 0.58, respectively, *Figure* 5I). In male EDL muscle, there was a quadratic trend noted in p62 protein content (*P* = 0.001, *Figure* 5O and 5L). In female EDL muscle, there was also a quadratic trend noted in p62 content (*P* < 0.001, *Figure* 5O and 5L). In male gastrocnemius muscle, there was a cubic trend in p62 protein content (*P* = 0.003, *Figure* 5P and 5L). In female gastrocnemius muscle, a quadratic trend was noted (*P* < 0.001, *Figure* 5P and 5L).

## Discussion

To our knowledge, this is one of the first studies to investigate mitochondrial quality in both male and female mice during early development and progression of disuse atrophy. We find muscle loss occurs quickly in both sexes; however, mitochondrial degeneration occurs prior to atrophy in male mice but after muscle atrophy in female mice. This observation suggests female mice protect mitochondrial health and quality control during the onset of disuse atrophy, and mitochondrial aberrations preceding muscle loss may be specific to male mice. More so, differences also appear to occur depending on the fibre type distribution of the muscle during disuse atrophies, with oxidative muscles displaying the greatest aberrations compared with glycolytic muscles.

Similar to prior works, we find robust muscle loss with hindlimb unloading in both male and female mice.^17^, ^22^, ^23^ Of note, we observe mitochondrial stress, measured by pMitoTimer, in male mice before the onset of detectible muscle loss.^10^ Paradoxically, male mice either had no changes in ROS production (gastrocnemius) or increased ROS production was only detectable after 7 days of disuse (soleus). Contrastingly, female mice had no changes to mitochondrial stress, but significant inductions of ROS production at 24 h of unloading in both gastrocnemius and soleus. These data suggest that mitochondrial oxidative stress and overall ROS production are not as tightly coordinated as previously believed. Moreover, the current data imply that male mice may not exhibit dramatic induction of ROS generation in the first few days of unloading; however, male mice do present considerable mitochondrial stress, similar to prior observations in cancer cachexia and other myopathies.^12^, ^13^, ^14^, ^24^ This mitochondrial stress precedes muscle loss in male mice, which may suggest a contributory role in the development of disuse‐atrophy. Conversely, in female mice, acute induction of ROS production at 24 h is not sufficient to alter mitochondrial stress, but may contribute to the activation of degradative pathways noted in our previous study using these same animals.^10^

Our recent works have found that male and female mice are differentially impacted by disuse interventions.^25^ However, in our prior study, mitochondrial‐targeted catalase protected mitochondrial quality and subsequent muscle mass in *female* mice, not male mice.^25^ Given the data in the current study, it is surprising that a mitochondria‐based intervention protected female mice, not male mice, especially considering alterations to mitochondrial health do not occur until after muscle loss in female mice (e.g. 168 h of disuse). We hypothesize that protections noted in the prior study may be related to increased ROS production in female mice after 24 h of disuse. Specifically, the initial ROS production within 24 h of disuse induces cellular signalling necessary to protect mitochondrial quality (such as mitophagy, described further later). This induction of mitochondrial quality signalling is then sufficient to maintain mitochondrial health during the initial development of disuse, but at the cost of muscle mass. Conversely, when female mice are given an intervention to mitigate ROS production, the energy necessary to maintain mitochondrial quality is shifted to conserving muscle mass. This hypothesis would reconcile the apparently contradictory data in this study and prior works^25^; however, more work is necessary to directly test this hypothesis.

Interestingly, we did not find alterations in RCR, a measure of ATP production, in either sexes or muscle groups across any time points. No change in RCR implies alterations to mitochondrial respiratory function and subsequent oxidative ATP production do not contribute to the early development of disuse‐induced muscle loss. However, this is perhaps unsurprising as our prior work demonstrated that alterations to RCR (in male mice) occur after the development of muscle atrophy.^16^ Whereas alterations to pMitoTimer‐measured oxidative stress and enhanced ROS production occur prior to the initial development of cancer cachexia.^16^ Taken together, these data likely suggest that reductions in mitochondrial ATP production occur after the initiation of muscle pathologies, if such reductions indeed manifest. More so, prior works in disuse atrophy demonstrate robust mitochondrial aberrations at 7 + days of unloading.^17^ As such, it is quite possible that the duration of unloading in our study was not sufficient to result in alterations to mitochondrial ATP production, implying that reduced ATP production is the result of disuse‐induced muscle loss, not necessarily the cause.

One aspect of mitochondrial quality control, which appeared consistently altered across the muscle types, was mitochondrial mRNA translation, the mRNA translation of genes encoded by mitochondrial genome that includes key components of the electron transport system.^26^ In the present study, we find reduced mRNA content of mitochondrial mRNA translation mechanisms across different muscle types. The most atrophy‐prone muscle (soleus) had the most consistent and robust reduction in mRNA markers of mitochondrial translation. These findings suggest that diminished translational capacity in the soleus and aligns with prior works from our laboratory in hindlimb unloaded rats.^27^ While speculative, within highly oxidative fibres, reductions in mitochondrial translation may strongly influence initial muscle wasting. Prior works have found that mitochondrial translation is tightly coordinated with mitochondrial biogenesis and subsequent mitochondrial quality control.^28^ However, precise mechanisms connecting mitochondrial translation to catabolic processes have not been established and require further investigation.

Although many aspects of mitochondrial dynamics appeared to change during the progression of atrophy, these alterations were not consistent across muscle fibre types. The notable exception to this finding is the decrease in *Opa1* mRNA content in the soleus muscle, which was diminished within 24 h in both male and female mice. Recent works have begun to elucidate the specific role for OPA1 in a variety of muscle pathologies.^29^ For example, OPA1 is also lower in murine models of cancer cachexia^16^ as well as disuse.^30^ More so, knockdown of OPA1 or alterations to OPA1 structure are sufficient to result in oxidative stress and muscle loss.^29^ Therefore, it is possible that reductions in OPA1 represent a common aberration in the progression of muscle loss across a variety of pathologies; however, this hypothesis requires further investigation.

In female mice, there was greater content of many makers of mitochondria fission such as MFF and FIS1 across multiple muscles. Based on our pMitotimer data, whereby red/green ratio was greater in male mice at 24 h (concurrent with greater *Mff* mRNA content in male soleus muscle) and at 168 h in female mice (also concurrent with greater *Mff* mRNA content in female soleus muscle) it appears more likely alterations in *Mff* mRNA content within the soleus are pathological and correlates with mitochondrial quality; although these findings would need further validation from additional studies.

Concurrent with dramatic muscle mass loss, female mice appeared to have changes in moderators of autophagy and mitophagy. These alterations coincide with prior works demonstrating increased autophagy and mitophagy in murine models of disuse atrophy.^22^, ^31^ Specifically, we find 4–10‐fold greater *Bnip3* in female mice unloaded for 24–48 h compared with male mice whom had marginal differences in *Bnip3* between unloading conditions. Many prior works have found increases in BNIP3 during the early phases of muscle atrophy,^32^, ^33^, ^34^ which then appears to reverse to decreased BNIP3 with prolonged durations (>2 weeks) of disuse.^27^ Yet to our knowledge, no study has included markers of mitophagy/autophagy content in both male and female rodents during these early phases of disuse. Speculatively, in female mice, these greater inductions of mitophagy may serve as a protective factor for the muscle, whereby increased mitophagy allows for early removal of unhealthy/damaged mitochondria and maintenance of a healthy mitochondrial pool, which would correspond with pMitoTimer and ROS data herein. Of note, this greater mitophagy in female mice also corresponds to the increase in ROS production noted in female mice within 24 h of disuse. This small ROS generation from the mitochondria potentially serves as a signalling mechanism for mitophagy, which in turn facilitates the maintenance of a healthier mitochondrial pool during the initial development of disuse in female mice compared with male mice (preserved red:green ratio).

Additionally, it is noteworthy that male and female mice appeared to have different inductions of general autophagy, specifically *Lc3* and *p62* during the initial days of disuse atrophy. Across the EDL, gastrocnemius, and soleus muscle female mice appeared to have greater inductions of autophagy, aligning with our prior work implying female mice have greater ubiquitin proteasome degradative responses to disuse.^10^ Overall, these works imply female mice have greater capacity for protein degradative responses compared with male mice during disuse‐atrophy, similar to prior works implying female mice have greater markers for autophagy compared with male mice at baseline.^35^, ^36^ Although how these potential differences relate to phenotypical outcomes is still not clear. Previous works have clearly established the role of autophagy on muscle loss during disuse atrophy using male or female models.^37^, ^38^, ^39^ However, to our knowledge, this is the first study to demonstrate sex differences in autophagy‐related alterations during disuse atrophy. Although we should acknowledge we are basing our conclusions primarily on mRNA content of mitophagy and autophagy moderators, and did not directly measure the mitophagy process itself.

**Figure 6:**
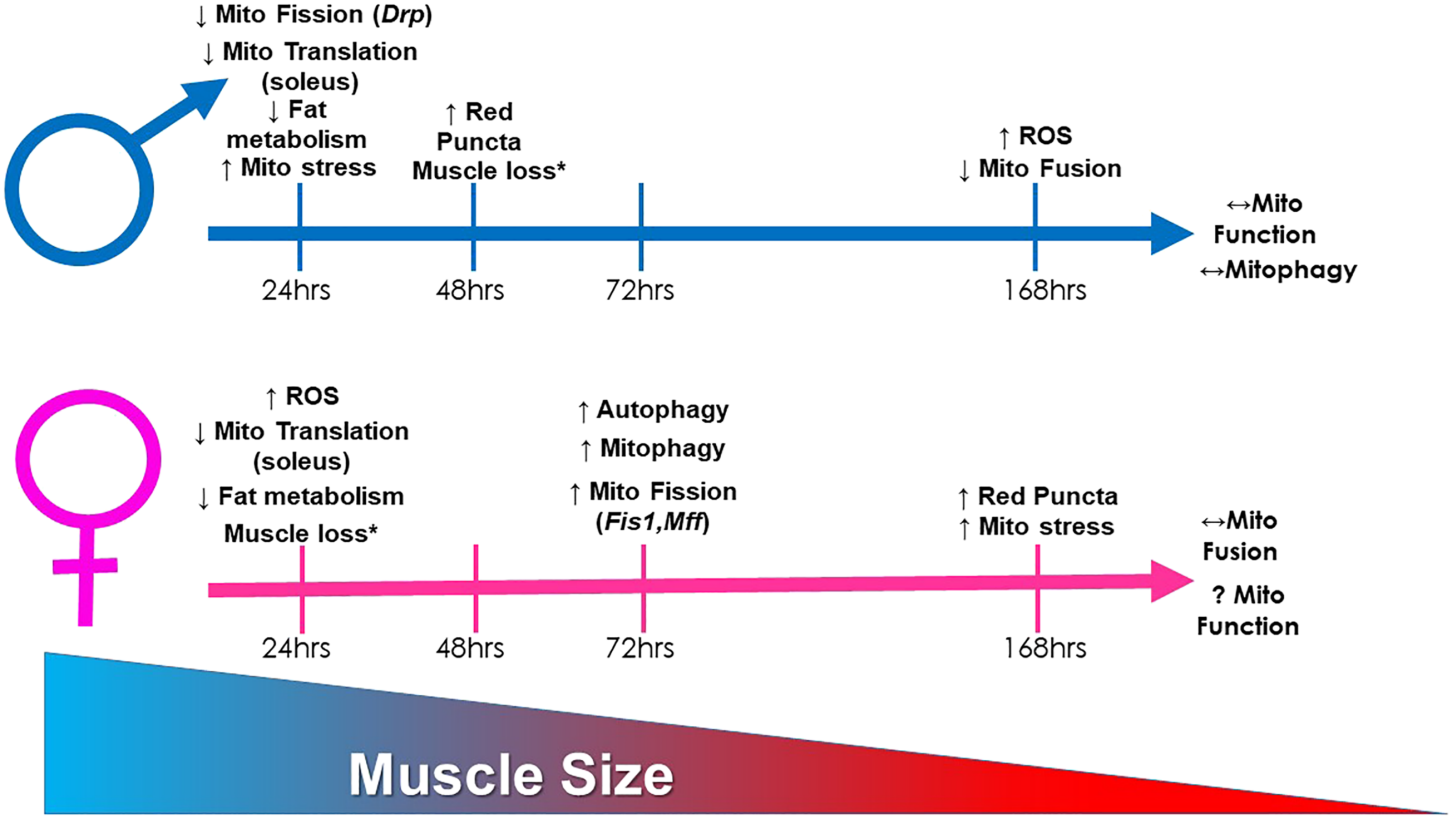
Pictorial representation of primary findings of the current study. * indicates data that have been previously reported.^10^

In conclusion, we find that even a relatively short bout of hindlimb unloading (24–48 h) is sufficient to result in muscle loss in both male and female mice as well as multiple aberrations to mitochondrial quality control (*Figure* 6). However, these aberrations occur on different time scales between male and female mice and may influence potential therapeutics to treat disuse. Interestingly, after 7 days of disuse, many aspects of male and female atrophy appear to ‘normalize’, and future works should investigate if sex differences remain throughout longer durations of disuse. We should acknowledge we were not able to directly statistically compare trends between male and female mice. As such, until more robust statistical methods are developed to test differential trends, these conclusions of differential progressions do warrant some uncertainty. While speculative, female mice's protection of mitochondrial quality early in disuse suggests female mice may preferentially protect metabolic health in lieu of muscle mass. This study provides a comprehensive evaluation of mitochondrial alterations during the development and progression in disuse atrophy across multiple muscle groups of varying fibre type in both male and female mice, providing an indispensable resource for the study of disuse‐induced muscle atrophy.

## Conflicts of interest

Megan E. Rosa‐Caldwell, Seongkyun Lim, Wesley A. Haynie, Jacob L. Brown, Kirsten Dunlap, Lisa T. Jansen, David E. Lee, Michael P. Wiggs, Tyrone A. Washington, and Nicholas P. Greene declare that they have no conflict of interest.

## Supporting information


**Figure S1.** SDH fiber distributions and Periodic acid–Schiff (PAS) staining data. **A.)** Male SDH + fiber frequency distribution. **B.)** Female SDH + fiber frequency distribution. **C.)** Male SDH‐ fiber frequency distribution. **D.)** Female SDH‐ fiber frequency distribution. **E.)** Glycogen content measured by PAS staining and associated representative images. Different letters indicate statistical differences within a sex at *p* < 0.05. * indicates linear trend, Ω indicates quadratic trend, # indicates cubic trend within as sex.

**Figure S2.** COXIV mitochondrial content data. **A.)** *Cox4* mRNA content in the EDL muscle. **B.)** *Cox4* mRNA content in the gastrocnemius muscle. **C.)** *Cox4* mRNA content in the Soleus muscle. **D.)** COXIV protein content in the EDL muscle. **E.)** COXIV protein content in the gastrocnemius muscle. **F.)** Representative images of COXIV western blot data. Different letters indicate statistical differences within a sex at *p* < 0.05. * indicates linear trend, Ω indicates quadratic trend, # indicates cubic trend within as sex.

**Figure S3** mRNA and protein markers of metabolism and mitochondrial biogenesis in the EDL, Gastrocnemius and Soleus muscles. **A.)** *Pparα* mRNA content in the EDL muscle in males and females. **B.)** *Pparα* mRNA content in the gastrocnemius muscle in males and females. **C.)** *Pparα* mRNA content in the soleus muscle in males and females. **D.)** *Nrf2* mRNA content in the EDL muscle in males and females**. E.)** *Nrf2* mRNA content in the gastrocnemius muscle in males and females. **F.)** *Nrf2* mRNA content in the soleus muscle in males and females. **G.)** *Pgc1α* mRNA content in the EDL muscle in males and females. **H.)** *Pgc1α* mRNA content in the gastrocnemius muscle in males and females. **I.)** *Pgc1α* mRNA content in the soleus muscle in males and females. **J.)** PGC1α protein content in the EDL muscle in males and females. **K.)** PGC1α protein content in the gastrocnemius muscle in males and females. **L.)** Representative images from the EDL and gastrocnemius muscles. Different letters indicate statistical differences within a sex at *p* < 0.05. * indicates linear trend, Ω indicates quadratic trend, # indicates cubic trend within as sex.

**Figure S4.** Western blot data for markers of mitochondrial fusion and fission. **A.)** MFN1 protein content in the EDL muscle in males and females. **B.)** MFN1 protein content in the gastrocnemius muscle in males and females. **C.)** MFN2 protein content in the EDL muscle in males and females**. D.)** MFN2 protein content in the gastrocnemius muscle in males and females. **E.)** OPA1 protein content in the EDL muscle in males and females. **F.)** OPA1 protein content in the gastrocnemius muscle in males and females. **G.)** Representative images of Western Blot images in the EDL and gastrocnemius muscles. **H.)** FIS1 protein content in the EDL muscle in males and females. **I.)** FIS1 protein content in the gastrocnemius muscle in males and females. **J.)** DRP1 protein content in the EDL muscle in males and females. **K.)** DRP1 protein content in the gastrocnemius muscle in males and females. **L.)** Representative images of Western Blot images from the EDL and gastrocnemius. Different letters indicate statistical differences within a sex at *p* < 0.05. * indicates linear trend, Ω indicates quadratic trend, # indicates cubic trend within as sex.

**Figure S5.** Additional LC3 quantification**. A.)** LC3II content in the EDL muscle. **B.)** LC3II content in the gastrocnemius muscle. **C.)** Total LC3 content in the EDL muscle. **D.)** Total LC3 content in the gastrocnemius muscle. Different letters indicate statistical differences within a sex at *p* < 0.05. * indicates linear trend, Ω indicates quadratic trend, # indicates cubic trend within as sex.

**Data S1.** Supporting information.

**Data S2.** Supporting information.
