# Supplementary figures and images for: Mitochondrial aberrations during the progression of disuse atrophy differentially affect male and female mice
Source: J Cachexia Sarcopenia Muscle. 2021 Sep 29;12(6):2056–68. doi: 10.1002/jcsm.12809 (PMC8718086; doi:10.1002/jcsm.12809)

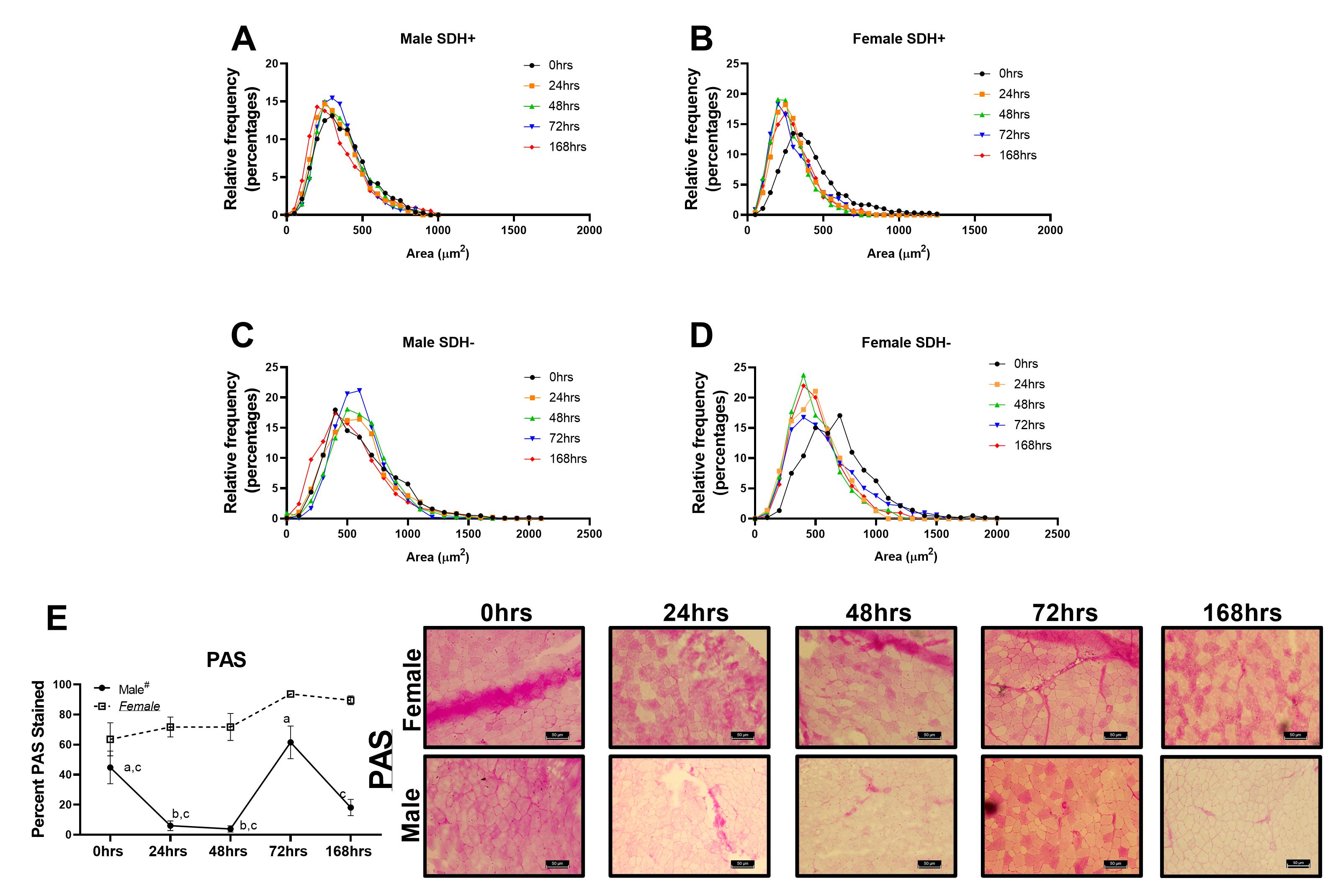

Supplement: Supplementary file 1 — Figure S1. SDH fiber distributions and Periodic acid–Schiff (PAS) staining data. A.) Male SDH + fiber frequency distribution. B.) Female SDH + fiber frequency distribution. C.) Male SDH‐ fiber frequency distribution. D.) Female SDH‐ fiber frequency distribution. E.) Glycogen content measured by PAS staining and associated representative images. Different letters indicate statistical differences within a sex at p < 0.05. * indicates linear trend, Ω indicates quadratic trend, # indicates cubic trend within as sex. [file JCSM-12-2056-s007.jpg]

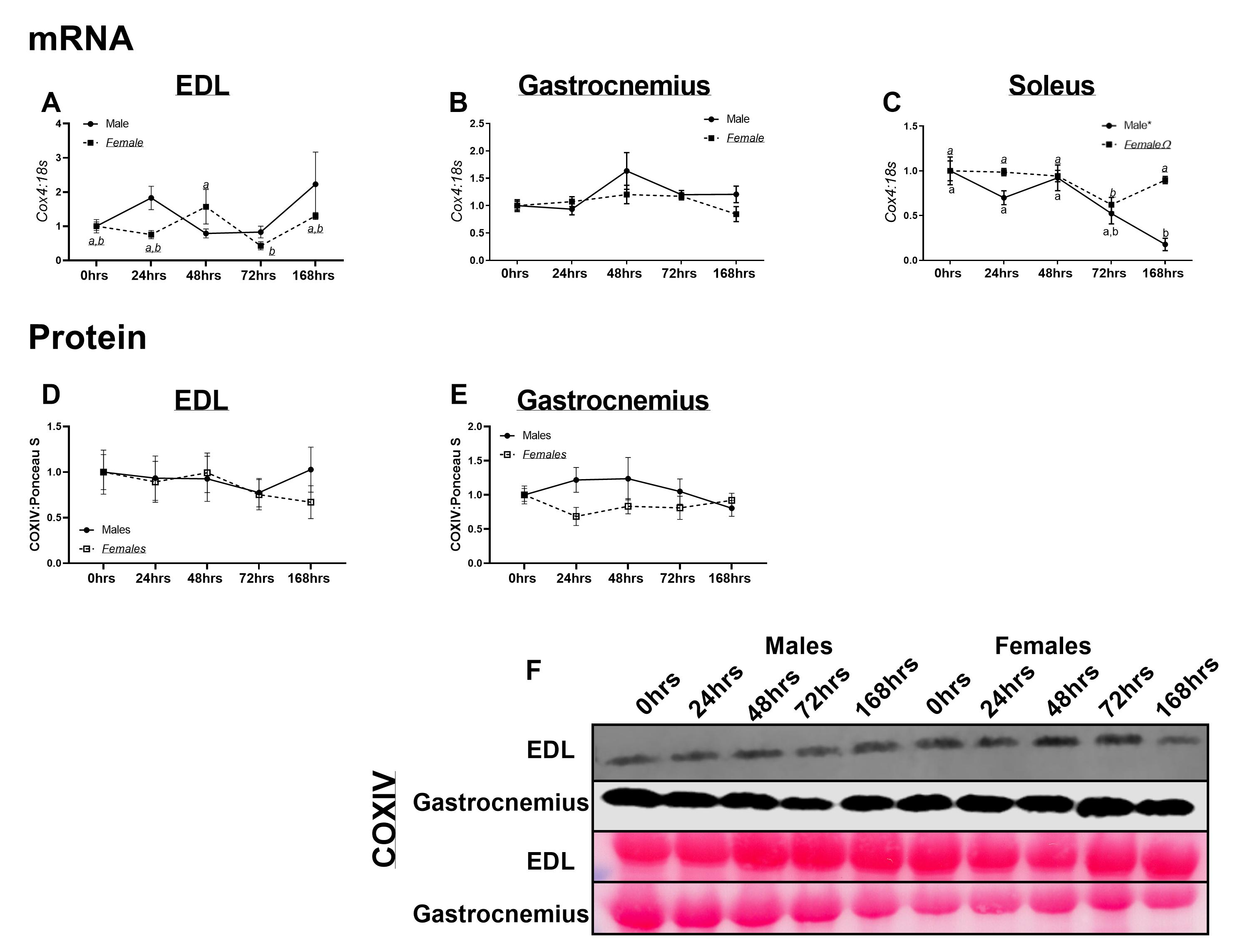

Supplement: Supplementary file 2 — Figure S2. COXIV mitochondrial content data. A.) Cox4 mRNA content in the EDL muscle. B.) Cox4 mRNA content in the gastrocnemius muscle. C.) Cox4 mRNA content in the Soleus muscle. D.) COXIV protein content in the EDL muscle. E.) COXIV protein content in the gastrocnemius muscle. F.) Representative images of COXIV western blot data. Different letters indicate statistical differences within a sex at p < 0.05. * indicates linear trend, Ω indicates quadratic trend, # indicates cubic trend within as sex. [file JCSM-12-2056-s002.jpg]

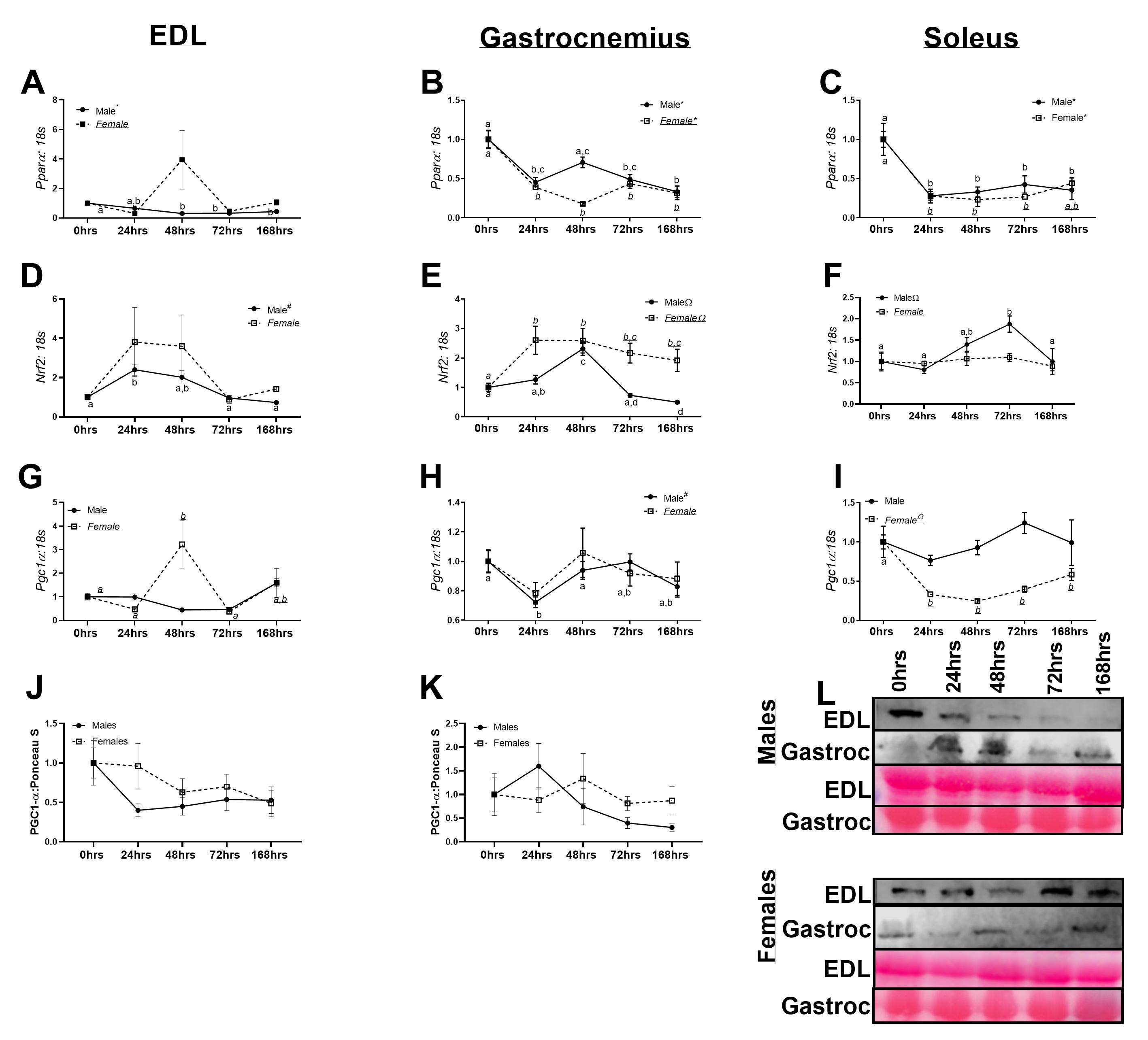

Supplement: Supplementary file 3 — Figure S3 mRNA and protein markers of metabolism and mitochondrial biogenesis in the EDL, Gastrocnemius and Soleus muscles. A.) Pparα mRNA content in the EDL muscle in males and females. B.) Pparα mRNA content in the gastrocnemius muscle in males and females. C.) Pparα mRNA content in the soleus muscle in males and females. D.) Nrf2 mRNA content in the EDL muscle in males and females. E.) Nrf2 mRNA content in the gastrocnemius muscle in males and females. F.) Nrf2 mRNA content in the soleus muscle in males and females. G.) Pgc1α mRNA content in the EDL muscle in males and females. H.) Pgc1α mRNA content in the gastrocnemius muscle in males and females. I.) Pgc1α mRNA content in the soleus muscle in males and females. J.) PGC1α protein content in the EDL muscle in males and females. K.) PGC1α protein content in the gastrocnemius muscle in males and females. L.) Representative images from the EDL and gastrocnemius muscles. Different letters indicate statistical differences within a sex at p < 0.05. * indicates linear trend, Ω indicates quadratic trend, # indicates cubic trend within as sex. [file JCSM-12-2056-s005.jpg]

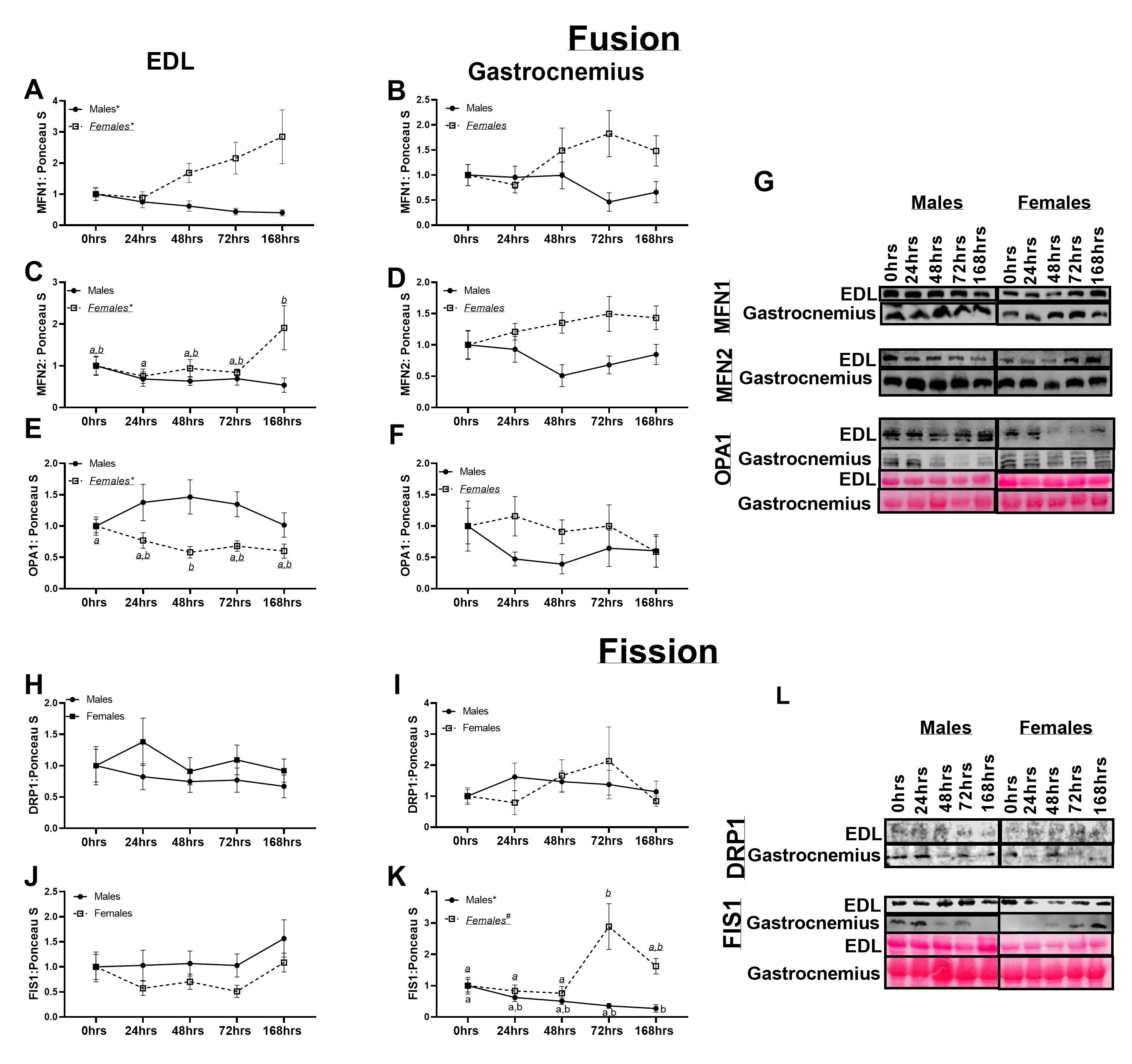

Supplement: Supplementary file 4 — Figure S4. Western blot data for markers of mitochondrial fusion and fission. A.) MFN1 protein content in the EDL muscle in males and females. B.) MFN1 protein content in the gastrocnemius muscle in males and females. C.) MFN2 protein content in the EDL muscle in males and females. D.) MFN2 protein content in the gastrocnemius muscle in males and females. E.) OPA1 protein content in the EDL muscle in males and females. F.) OPA1 protein content in the gastrocnemius muscle in males and females. G.) Representative images of Western Blot images in the EDL and gastrocnemius muscles. H.) FIS1 protein content in the EDL muscle in males and females. I.) FIS1 protein content in the gastrocnemius muscle in males and females. J.) DRP1 protein content in the EDL muscle in males and females. K.) DRP1 protein content in the gastrocnemius muscle in males and females. L.) Representative images of Western Blot images from the EDL and gastrocnemius. Different letters indicate statistical differences within a sex at p < 0.05. * indicates linear trend, Ω indicates quadratic trend, # indicates cubic trend within as sex. [file JCSM-12-2056-s003.jpg]

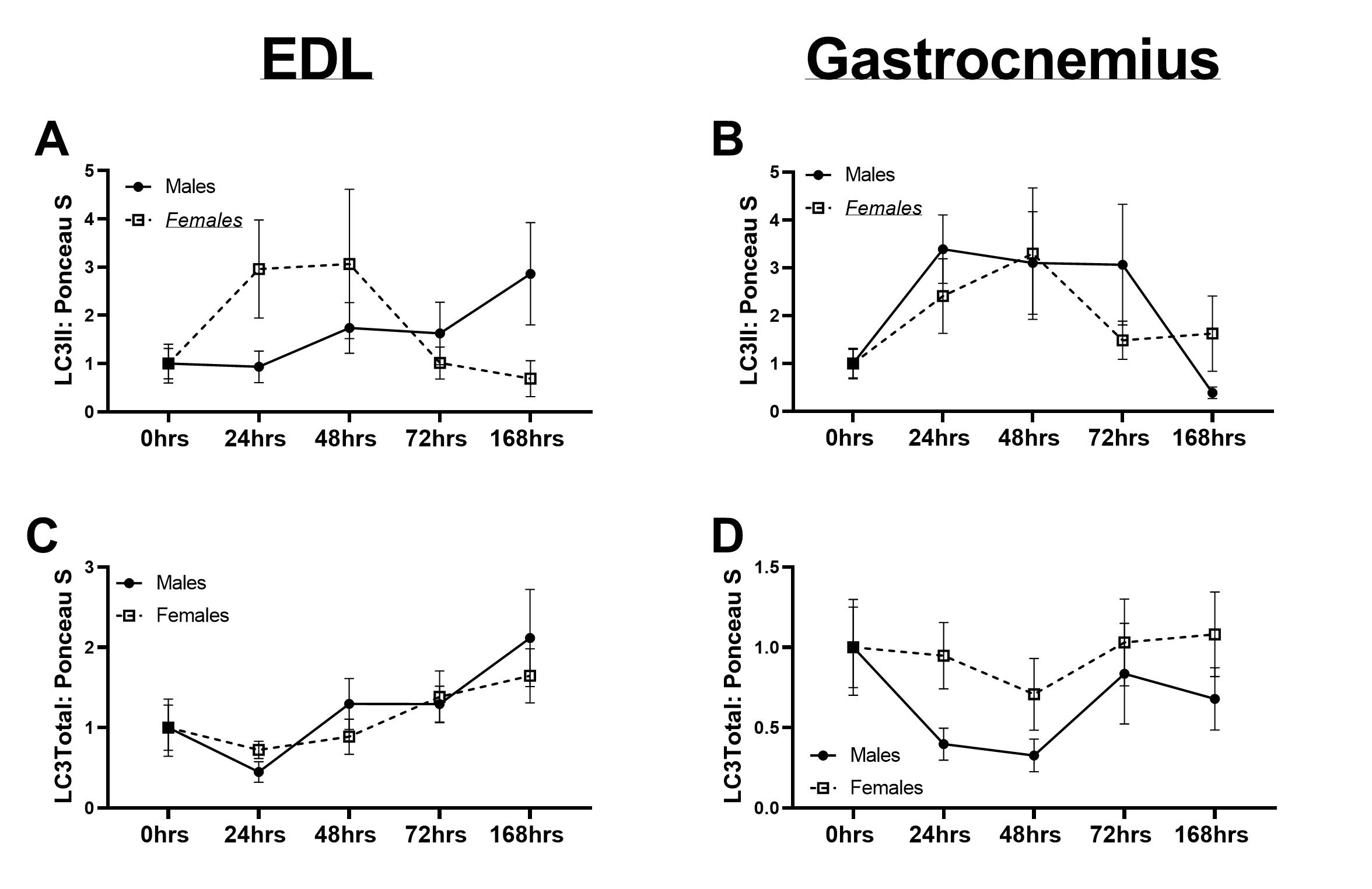

Supplement: Supplementary file 5 — Figure S5. Additional LC3 quantification. A.) LC3II content in the EDL muscle. B.) LC3II content in the gastrocnemius muscle. C.) Total LC3 content in the EDL muscle. D.) Total LC3 content in the gastrocnemius muscle. Different letters indicate statistical differences within a sex at p < 0.05. * indicates linear trend, Ω indicates quadratic trend, # indicates cubic trend within as sex. [file JCSM-12-2056-s006.jpg]
