# Supplementary material for: Mitochondrial aberrations during the progression of disuse atrophy differentially affect male and female mice
Source: J Cachexia Sarcopenia Muscle. 2021 Sep 29;12(6):2056–68. doi: 10.1002/jcsm.12809 (PMC8718086; doi:10.1002/jcsm.12809)
Supplement: Supplementary file 6 — Data S1. Supporting information. [file JCSM-12-2056-s004.docx]

**Supplementary Methods**

Hindlimb Unloading

Animals’ tails were first sterilized using ethanol wipes and iodine swabsticks. Then animals’ tails were coated with benzoin tincture solution. After which, athletic tape was wrapped around the animals’ tails. Tails were then suspended through a specialized hook apparatus attached to the athletic tape. This apparatus allowed for animals to freely move about the cage with their forelimbs with the hindlimb remaining elevated from the floor of the cage. Animals were monitored daily to ensure continuous unloading as well as to monitor for signs of tail necrosis, or distress.

pMitoTimer Electroporation

Briefly, animals were anesthetized with 2% isoflurane mixed with oxygen. Upon anesthesia, 10 µL of of hyaluronidase solution (0.36mg/ml dissolved in sterile saline) was injected into the right FDB of each mouse. After one hour animals were re-anesthetized and the right FDBs were injected subcutaneously with 20 µg of pMitoTimer dissolved in 10 µL sterile saline. Animals were allowed to recover for 10 minutes, then the FDB underwent electroporation with 10 pulses at 75 V/cm, 1Hz and 20ms/pulse.

The FDB muscle was chosen for this analysis for a few reasons. 1.) Due to the anatomy of the muscle, it can be whole mounted directly on a microscope slide and analyzed within an hour of tissue harvest. 2.) the FDB is responsive to atrophic stimuli^1–3^ and 3.) Due to the nature of the model the FDB was concurrently unloaded along with other hindlimb muscles.

pMitoTimer Histological Quantification

Briefly, flexor digitorum brevis (FDB) muscles were excised at tissue harvest and fixed in 4% paraformaldehyde/PBS solution for 20 minutes. After which, FDBs were washed for 5 minutes in PBS and mounted on a gelatin coated slide. Slides were then analyzed for red and green fluorescence using FITC and TRITC filters on a Nikon Ti‐S inverted epiflourescent microscope (Melville, NY) with LED‐based light source with tightly controlled acquisition parameters^2,3^. All images presented were acquired at 100X magnification with an objective resolution of 220nm. Acquisition parameters (such as exposure time) were determined through pilot studies to ensure exposure times were sufficient to detect red and green fluorescence without saturating the image. All images were acquired within 1-2 hours of tissue harvest.

For image analysis, each image was divided into red and green florescent JPEG images with the same image parameters (intensity, exposure etc.) for all images across all groups. Images were then processed to create gray-scale images using open access Cell Profiler Software^4^. Processed images were then analyzed using a specially designed MatLab program^2^, a generous gift of Zen Yang. Data were analyzed by Red/Green ratio, with a greater Red/Green ratio indicating mitochondrial stress, and number of red puncta, with greater red puncta content associated with greater degenerated mitochondria. Our Red/Green ratio and red puncta data were on a scale comparable to our previous works and the original works validating this measure^2,3^.

### Mitochondrial Respiration

Mitochondrial respiration was completed as previously described^1^. For this measure we used the entire soleus and a portion of the mixed fibers from the gastrocnemius. Briefly, soleus and gastrocnemius muscles were excised from live animals and then carefully teased into near individual muscle-fibers bundles. Muscle fibers were teased apart in ice-cold buffer X containing 60 mm K-Mes, 35 mm KCl, 7.23 mm K_2_EGTA, 2.77 mm CaK_2_EGTA, 20 mm imidazole, 0.5 mm dithiothreitol, 20 mm taurine, 5.7 mm ATP, 15 mm PCr and 6.56 mm MgCl_2_, pH 7.1). Fibers were then permeabilized in saponin dissolved in buffer X (0.5mg/mL) on a tube rocker kept at 4^0^ C for 30 minutes. Afterwards, permeabilized fibers were washed with ice-cold buffer Z (containing 10 mm K-Mes, 35 mm KCl, 1 mm EGTA, 5 mm K2HPO4, and 3 mm MgCl2, 0.005 mm glutamate, 0.02 mm malate and 0.5 mg ml−1 BSA, pH 7.1).

Mitochondrial oxygen consumption of the fibers was measured polargraphically (Oxygragph, Hansatech Instruments, Norfolk England). In the presence of malate and glutamate ( 5mM and 5 mM respectively) , mitochondrial respiration was measured in ADP-stimulated (0.25 mM, state 3) and oligomyin-inhibited respiration (10 µg/mL, LEAK/state 4).

After measurement of oxygen consumption, tissues were frozen in liquid nitrogen. At a later date, tissues were dried to remove all water. Dried tissues masses were measured and respiration values were normalized to dry tissue weights. Respiration was normalized to dry tissue weights and is presented as nmole/min*mg.

### Reactive Oxygen Species Production

Briefly, permeabilized fibers from soleus and gastrocnemius muscle were incubated in respiration buffer along with with Amplex Ultra Red (AR) (Thermo Fisher Scientific) . The reaction of H_2_O_2_ and AR is catalyzed by horseradish peroxidase to produce the red fluorescent compound resorufin (excitation wavelength 563 nm, emission 587 nm). The change of emitted fluorescence intensity is directly proportional to the concentration of H_2_O_2_ Fluorescence was quantified using Maya LSL Spectrometer (Ocean Optics) with the appropriate excitation source. Baseline fluorescence (respiration buffer, permeabilized fibers, HRP, and AR) was assessed for 8 minutes and then H_2_O_2_ production was increased by the addition of succinate (final concentration of 10 mM). The slopes at baseline and succinate-stimulated were measured, converted to H_2_O_2_ concentrations based on an H_2_O_2_ -derived standard curve and corrected by dry tissue mass.

Histological Quantification

#### Succinate Dehydrogenase Stain: Succinate dehydrogenase (SDH) stain was completed in 10 µm sections of the tibialis anterior muscle as we have previously described^3^. Muscle fibers were dichotomized into either SDH positive (SDH+) or SDH negative (SDH-) fibers. Fiber cross sectional area (CSA) for SDH+ and SDH- fibers was assessed as we have previously described^3^. Male and female cross sectional area for both SDH+ and SDH- fibers were averaged within each animal.

*Periodic acid–Schiff stain:* Periodic acid–Schiff stain (PAS) stain was completed on 10 µm sections of the tibialis anterior muscle using similar methods as previously described^5^. Images were analyzed for area of stain using Nikon NIS-Elements Basic Research Software (Nikon Instruments Inc., Melville, NY). All images for each animal were averaged and expressed as percent area stained as previously reported^5^.

mRNA Quantification

Muscles were homogenized in TriZOL reagent to extract RNA. RNA was extracted and reverse transcribed into cDNA as previously described^6^. cDNA was analyzed using SYBR Green primer pairs or Taqman probes as appropriate using the ^-ΔΔ^CT method as we have previously described^6^. All samples were analyzed using a StepOne PCR instrument (ThermoFisher Scientific, Waltham, MA). SYBR primers included: *Pparα, Nrf2, Mfn1, Mfn2, Opa1, Drp1, Fis1, Mff, mtiF2, mtiF3, Taco1, Tufm, Tfam,* and *Bnip3* (all primer pairs have previously been reported^6^. Taqman probes included: *18s* (Clone #Mm03928990_g1), *Cox4* (Clone #mm01250094_m1), *Pgc1α* (Clone # mm01208835_m1), *Lc3* (Clone # Mm00458725_g1) and *p62* (Clone #Mm00448091_m1).

Western blot

All samples were homogenized, protein concentration quantified, run and transferred on PVDF membranes and incubated in primary and secondary antibody solutions as we have previously reported^6^. All membranes were imaged using a LiCor Odyssey® Fc Imaging System and analyzed using ImageStudio Lite software (LiCor, Lincoln, NE). For all membranes, one cohort of males (0hr, 24hr, 48hr, 72hr, and 168hr) and one cohort of females (0hr, 24hr, 48hr, 72hr, and 168hr) was loaded on each membrane. To compare samples across gels an internal control sample was loaded on each membrane and samples were normalized to Ponceau S stain as loading control and the internal control.

**References**

1. Min K, Kwon O-S, Smuder AJ, Wiggs MP, Sollanek KJ, Christou DD *et al.* Increased mitochondrial emission of reactive oxygen species and calpain activation are required for doxorubicin-induced cardiac and skeletal muscle myopathy. *J Physiol* 2015;**593**:2017–2036.

2. Laker RC, Xu P, Ryall KA, Sujkowski A, Kenwood BM, Chain KH *et al.* A novel MitoTimer reporter gene for mitochondrial content, structure, stress, and damage in vivo. *J Biol Chem* 2014;**289**:12005–12015.

3. Brown JL, Rosa‐Caldwell ME, Lee DE, Blackwell TA, Brown LA, Perry RA *et al.* Mitochondrial degeneration precedes the development of muscle atrophy in progression of cancer cachexia in tumour‐bearing mice. *J Cachexia Sarcopenia Muscle* 2017;**8**:926–938.

4. McQuin C, Goodman A, Chernyshev V, Kamentsky L, Cimini BA, Karhohs KW *et al.* CellProfiler 3.0: Next-generation image processing for biology. *PLoS Biol* 2018;**16**:e2005970.

5. Rosa-Caldwell ME, Brown JL, Lee DE, Wiggs MP, Perry RA, Haynie WS *et al.* Hepatic alterations during the development and progression of cancer cachexia. *Appl Physiol Nutr Metab* 2020;**45**:500–512.

6. Greene NP, Lee DE, Brown JL, Rosa ME, Brown LA, Perry RA *et al.* Mitochondrial quality control, promoted by PGC-1α, is dysregulated by Western diet-induced obesity and partially restored by moderate physical activity in mice. *Physiol Rep* 2015;**3**.
