# Supplementary material for: Mitochondrial aberrations during the progression of disuse atrophy differentially affect male and female mice
Source: J Cachexia Sarcopenia Muscle. 2021 Sep 29;12(6):2056–68. doi: 10.1002/jcsm.12809 (PMC8718086; doi:10.1002/jcsm.12809)
Supplement: Supplementary file 7 — Data S2. Supporting information. [file JCSM-12-2056-s001.docx]

In male and female EDL muscle, there were no significant differences or pairwise comparisons in PGC1-α content (p=0.133 and 0.379 respectively Figure 3J and 3L). In male and female gastrocnemius muscle, there were no significant differences in PGC1-α content (p=0.083 and p=0.204 respectively, Figure 3K and 3L).

In male EDL muscle, there was a significant trend for decreased MFN1 content with prolonged periods of hindlimb unloading (p=0.010, Figure 4J and 4L); however, no pairwise differences reached statistical significance.

Within male EDL muscle, there was no significant trend or pairwise differences in MFN2 content (p=0.409, Figure 4M and 5L). However, in female EDL muscle, there was a significant linear trend for greater MFN2 content with longer durations of hindlimb unloading (p=0.005, Figure 5M and 5L), with 168hr females having 1.5-fold greater MFN2 compared to 24hr females. In addition, no differences were noted in MFN2 content in either male (p=0.405) or female (p=0 450, Figure 5N and 5L) gastrocnemius muscle.

In male EDL muscle there was no differences in OPA1 content (p=0.483, Figure 5O and 5L). However, in female EDL muscle, there was a linear trend (p=0.048, Figure 5O and 5L) with a progressive decrease in OPA1 with increased durations of unloading. The only pairwise difference noted as between 0hr and 48hr, with 48hr animals having ~50% less OPA1 compared to 0hrs (Figure 5O and 5L). In male gastrocnemius muscle, no differences or trends were noted in OPA1 content (p=0.142, Figure 5P and 5L). Similarly, females also had no differences noted in gastrocnemius OPA1 content (p=0.895, Figure 5P and 5L).

In both male and female EDL muscle, there were no significant trends or pairwise difference in DRP1 (p=0.834 and p=0.772 respectively, Figure 6J & 6N). In both male and female gastrocnemius muscle, there were no significant differences or pairwise comparisons in DRP1 content (p=0.804 and p=0.122 respectively, Figure 6L and 6N).

With regard to total LC3 content, there were no significant differences in total LC3 content noted in either males (p=0.116, Supplementary Figure 3) or females (p=0.847, Supplementary Figure 3).

In both male and female EDL muscle there were no significant trends or pairwise differences in FIS1 content (p=0.594 and p=0.191 respectively, Figure 6J & 6N). In male gastrocnemius muscle, there was a linear trend for decreased FIS1 content with prolonged duration of unloading (p=0.002, Figure 6K & 6N), with 168hr males having ~73% less FIS1 content compared to 0hr animals. Conversely, in female gastrocnemius muscle, a significant cubic trend was detected, with 72hr females having ~2-fold greater FIS1 content compared to 0hr, 24hr, and 48hr animals (p=0.0015, Figure 6K & 6N).
